# Ozone Efficiency on Two Coleopteran Insect Pests and Its Effect on Quality and Germination of Barley

**DOI:** 10.3390/insects13040318

**Published:** 2022-03-24

**Authors:** Xue Dong, Manjree Agarwal, Yu Xiao, Yonglin Ren, Garth Maker, Xiangyang Yu

**Affiliations:** 1 Institute of Agricultural Resources and Environment, Jiangsu Academy of Agricultural Sciences, Nanjing 210014, China; xue_dong1990@126.com; 2 College of Science, Health, Engineering and Education, Murdoch University, Perth 6150, Australia; m.agarwal@murdoch.edu.au (M.A.); amy.xiao@murdoch.edu.au (Y.X.); y.ren@murdoch.edu.au (Y.R.)

**Keywords:** fumigant, ozone (O_3_), barley (*Hordeum vulgare* L.), germination, insect control, quality

## Abstract

**Simple Summary:**

*Rhyzopertha dominica* (Fabricius) and *Tribolium castaneum* (Herbst) are notorious global pests, destroying various stored grains, including barley, wheat, oats, maize, and rice. Ozone (O_3_) is a promising fumigant to control pests in stored grain since it can safely and rapidly auto-decompose without leaving residues, however, relatively few studies have focused on the toxicity of O_3_ on stored grain pests in stored barley. In this study we not only explored the susceptibility of all life stages of *R. dominica* and *T. castaneum* in barley seeds to different durations of gaseous O_3_, but also investigated the effect of O_3_ on germination ability, seedling growth, and quality of barley. O_3_ was effective against all life stages of two species in barley under sufficient exposure times without negative impacts affecting the commercial quality of barley. However, the germination ability and seedling growth were adversely impacted at longer O_3_ exposure times. Thus, it is imperative to select an optimal O_3_ exposure time to achieve the desired functional outcome, such as malting, animal feeding, and human consumption.

**Abstract:**

Ozone (O_3_) is a potential fumigant to control pests in stored grain since it can safely and rapidly auto-decompose without leaving residues. In this study, the efficacy of O_3_ on all life stages of *Rhyzopertha dominica* (Fabricius) and *Tribolium castaneum* (Herbst) in barley and the physiological effects on barley and its quality were investigated. Complete control of all life stages of pests was obtained at 700 ppm for 1440 min of ozone exposure without negatively impacting the contents of soluble protein, moisture content, seed colour, hardness, and the weight of thousand barley seeds. The eggs and pupae of these two insects were the more tolerant stages than their larvae and adults. Prolonged exposure times (40 to 1440 min) and mortality assessment intervals (1, 2, and 7 days) increased O_3_ efficacy due to the reaction characteristics and delayed toxicity. Aging barley seeds appeared to be more sensitive to prolonged ozone duration than new seeds. A total of 20 and 40 min could promote germination rate, and longer O_3_ exposure (1440 min) was unfavourable for germination and seedling growth. Thus, it is imperative to select an optimal O_3_ exposure time to transfer ozone into quality contributors of final products and achieve the desired functional outcomes.

## 1. Introduction

Postharvest loss has become a crucial issue of the grain supply chain. It has been estimated that one-quarter to one-third of the world’s grain crop is lost every year during storage, with the majority attributed to insect attack [1]. The lesser grain borer, *Rhyzopertha dominica* (Fabricius), and the red flour beetle, *Tribolium castaneum* (Herbst), are notorious global pests, destroying various stored grains, including barley, wheat, oats, maize, and rice. Both species can cause severe economic loss by decreasing the quantity and quality of stored grain. As a primary grain insect, *R. dominica* has the ability to attack whole kernels, while *T. castaneum* is a secondary pest and can only attack damaged grain, dust, and milled products [2]. Fumigation is often the cheapest and most effective process that is available, playing a major role world-wide in preserving commodities [3]. Therefore, currently, fumigation is a common method to eliminate grain storage insect pests. In actual practice, however, most gases have been eliminated owing to unfavourable properties, the most important being chemical instability and destructive effects on commodities [4]. Methyl bromide is a fast-acting fumigant, but it has been removed from fumigant registration because it caused depletion of ozone layer. Phosphine is the widely used fumigant, however, continuous use has led to the evolution of resistant populations and environmental contamination [5]. *R. dominica* was the first pest with strong resistance to phosphine recorded in Australia [6]. Nayak analyzed 20 years of resistance data in Australia, suggesting that resistance to phosphine had increased significantly over this period and is currently between 60–80% (depending on species). In North America, there was a significant increase in the frequency of resistance in both *T. castaneum* and *R. dominica* over two decades [7,8]. Opit suggested that the most resistant *T. castaneum* population was 119-fold more resistant than the susceptible strain and the most resistant *R. dominica* population was over 1500-fold more resistant in North America [9]. Strongly resistant populations of *T. castaneum* and *R. dominica* were also reported in Asia, including China [10,11], Pakistan [12], and India [13,14]. Ethyl formate is an alternative one, but it has high water solubility which could be rapid absorbed by grain, thus it just has been registered as a fumigant in dried fruit in Australia, not in grain industry [15]. Diatomaceous earths are used to control stored grain, causing insects death through absorbing the epicuticular lipids of the insect cuticle, but it has a negative effect on the physical properties of grains, particularly bulk density [16,17,18]. Controlled atmosphere with inert gases such as CO_2_ is an alternative non-chemical method to control grain pests, however, it requires long exposure times (more than 10 days) [19]. Entomopathogenic fungi (EF) are considered the most promising biocontrol agents, which infect insects through mycelium penetrating into the insects cuticle and then growing in the haemocoel causing insects death [20]. However, there are no commercial biopesticides based on EF bioagents that are registered against the stored-grain insects [20]. The main limitations of the application of EF in insect control are the high moisture requirement for conidial germination and subsequent sporulation, more time consuming to kill insects, and the potential risk to immunodepressive or immunocompromised people [21,22]. Other non-chemical methods such as cold or low temperature treatment and irradiation have also been reported to eliminate the insects in stored grain, however, they are costly for commercial treatment of large-scale bulk grain or need long-term treatment [23,24,25]. Therefore, alternative methods are needed to control grain storage pests.

Ozone (O_3_) displays distinct advantages which could overcome the deficiencies of other fumigants. O_3_ occurs naturally in the atmosphere and could be generated on-site without transportation problems. Ozone can rapid auto-decompose to oxygen without leaving residues, along with a wide spectrum of activity against microorganisms, making it an attractive approach for medical sterilisation, food processing, and grain storage [26,27,28]. There are more than 100 years of using ozone in food industry and it was firstly used as food preservative of frozen meat in 1910 [29]. In 2001, The United States Food and Drug Administration (FDA) approved O_3_ as a direct additive for food treatment, storage, and processing [30]. For fruit processing, Sadeghi et al. [31] applied ozone to control *Oryzaephilus surinamensis* (Coleoptera: Silvanidae) and *Ephestia kuehniella* (Lepidoptera: Pyralidae) in stored dried figs, reporting that increasing the ozone concentration and times had no significant influence on the quality parameters of dried figs and raisins, including colour, sweetness, sourness, brittleness, hardness, and general acceptance. Ozone is also effective at eliminating storage pests, microbes, and degrading mycotoxins in grain [32,33,34]. There has been growing emphasis on the application of O_3_ to control *R. dominica* and *T. castaneum*. Sousa et al. [35] reported that 150 ppm gaseous O_3_ presented a high efficacy against both susceptible and phosphine-resistant insects from 16 populations of *T. castaneum* and 11 populations of *R. dominica*. O_3_ was also lethal to different stages of *R. dominica* and *T. castaneum* [36,37]. O_3_ presented a different efficacy against insects inside or outside the grain, mainly because grains can increase the decomposition of O_3_ [27]. The reaction of O_3_ within a grain can be divided into two phases. The first phase is that O_3_ interacts with active sites that are present in or on the kernel surface, causing the decomposition of O_3_. The second phase is the free movement of O_3_ through the grain layers once the reactive sites are saturated, with O_3_ concentration gradually increasing to an effective dosage for target pests [38]. The penetration of ozone into the bulk of grains also depends on gas diffusion, air speed within the grain layer, initial ozone concentration, bed thickness, temperature, and adsorption by the grain surface [39,40].Various grains have distinct effects on O_3_ insecticidal efficacy, which is attributed to differences in the surface and size of kernels.

The successful application of O_3_ against stored grain insects requires sufficient concentration and exposure time, however it may affect the grain quality. Zhu [41] reviewed that moderate O_3_ treatment could facilitate the dough strength, enhance the storage stability, and the whiteness of wheat flour. The malting quality of barley, such as alpha-amylase, fine grind extract, dynamic viscosity, and soluble protein were not impacted by O_3_ treatment of 26 mg/g [42]. Conversely, as a strong oxidant, O_3_ can react with some substrates in grains directly or via producing reactive oxygen species. Starch and lipid oxidation, protein modifications, grain discolouration, odour alteration, and germination loss may result from excessive use of O_3_ [43]. 

There were discrepancies in the effect of O_3_ on grain quality when the dosage of O_3_ was sufficient for eliminating insects pests in grain [43,44]. These discrepancies might be mainly caused by the differences of dose, duration, and grains. While some studies have assessed the efficacy of O_3_ against pests in maize and wheat, relatively few studies have focused on the toxicity of O_3_ on stored grain pests with barley. Thus, this study aims to explore the susceptibility of all life stages of *R. dominica* and *T. castaneum* in barley seeds to different durations of gaseous O_3_, and to investigate the effect of gaseous O_3_ on germination ability, seedling growth, and quality of barley with different storage times. Additionally, the different response of old and new barley seeds that were subjected to ozone treatment was also examined.

## 2. Materials and Methods

### 2.1. Insect Rearing

There were two insect species of stored product insects that were used for bioassays, these were *Tribolium castaneum* (Herbst) (Coleoptera: Tenebrionidae) and *Rhyzopertha dominica* (F.) (Coleoptera: Bostrychidae). They were the phosphine-susceptible MUWTC-6000 and MUWRD-7 strains, respectively, that were held at the Postharvest Biosecurity and Food Safety Laboratory, Murdoch University, Australia. The narrow-aged insects (2–3 days) were obtained by incubating 3000 adult insects with 1000 g of food, broken wheat Australian Standard White (ASW) for *R. dominica* and wheat flour/yeast 12:1 ratio for *T. castaneum* in 2 L jars that were sealed with meshed lids. The parent insects were removed after 3 days and the remaining culture medium were incubated in 2 L glass jars at 28 ± 1 °C and 70 ± 2% relative humidity (RH) with a 0:24 (L:D) h photoperiod. The newly emerged adults were narrow aged and transferred to a 2 L jar containing fresh food. The insects that were used in the experiments were one month old.

### 2.2. Barley Samples

Barley samples belonging to *Sparticus* CL were harvested in 2013/2014 and 2019/2020 from Lake Grace, Western Australia. The sample that was harvested in 2013/2014 was stored at 4 °C for 6 years. In the grain production business, the long-term storage of grain often occurs due to the relatively limited demand for consumption, cultivation, unexpected meteorological disasters, and also to prevent a change in specific characters [45]. The moisture content of the 2013/2014 and 2019/2020 harvest barley were 12.3% and 10.9%, determined with a Graintec HE 50 electronic moisture meter (Graintec Pty Ltd., Toowoomba, Australia). The results that were obtained were calculated from four replicate measurements. The insect-free samples were stored at 4 °C and equilibrated to room temperature before germination tests and quality measurements were conducted to room temperature at 25 ± 1 °C.

### 2.3. Fumigation Procedures with Ozone

A commercial O_3_ generator (Model FH-CYJ1520A-20 g/h, Shanghai Fenghua Optoelectronics Technology Co. Ltd., Shanghai, China) was utilized to treat the barley seeds. This generator continuously produced 700 ppm O_3_ from atmospheric air. The ozonation of barley seeds was carried out in a 2.4 L and 16.6-cm diameter Hysil semi-batch reactor with a desiccation glass chamber that was fitted with a lid containing a 2-cm central hole for ozonation. The gas was introduced from the ozone generator directly to the bottom of the reactor containing 1 kg of barley and passed through the seeds using a 1-cm diameter plastic tubing. Prior to introducing the O_3_, the barley seeds (1 kg) were mixed with 3 g adults (about 2000) and 7 g egg. The pupae and larvae were placed on a supporting metal mesh with 13-cm diameter, and 5 cm from the bottom to enable gas circulation. The ozone concentration to and from the reactor was continuously measured by an ozone monitor (Shenzhen Yuan Technology Co., Ltd., Shanghai, China). A total of nine treatments were designed for this study, comprising of a control (fresh air pass through the glass chamber), 700 ppm O_3_ exposure for 0, 10, 20, 40, 120, 240, 480, 960, and 1440 min, with three replicates. All the treatments were performed in ambient conditions at 20 ± 5 °C and 55 ± 3% RH.

### 2.4. Assessment of Insect Mortality

For the bioassay samples, all the adults, larvae, pupae, and eggs of the treated and untreated controls were retrieved at the end of the fumigation period from the above-treated and untreated barley with 710 and 180 μm sieves. The adults were removed and placed into 250 mL vials with food and incubated at 28 ± 1 °C and 70 ± 2% RH. The live and dead adult insects were counted at different assessment intervals, including immediately (0DAT), 1, 2, and 7 days after O_3_ treatment (1DAT, 2DAT, 7DAT). The remaining mixed-age cultures were incubated at 28 ± 1 °C and 70 ± 2% RH. The subsequent emerging adult insects were counted weekly for a period of 5 weeks, with live and dead adults removed at each count. The mortality was calculated based on a comparison of the emerging adults between the treated and untreated control samples. Once all the control insects emerged, the experiment was terminated.

### 2.5. Germination Test

The germination test was carried out based on the between-paper (BP) method of the International Seed Testing Association methods [46]. A total of 424 seeds (eight replicates of 53 seeds) in each harvest year were randomly selected. A large filter paper was saturated with 60 mL distilled water and folded in half, creating a double thickness. A steel template (290 × 580 mm) was placed over this wet paper, with holes in this stencil allowing the barley seeds to be placed 30 mm apart. Once the seeds were positioned correctly, the template was removed and the upper half of the filter paper was folded over the seeded area. The folded paper containing the seeds was then loosely rolled from one side perpendicular to the base and tied with elastic bands. All the treatments were stored individually in polythene zip bags and incubated for 7 days at 25 ± 1 °C in the dark.

The germination rate was detected 7 days after the start of the germination test. The morphological characteristics were tested, including the length of the shoot, the length of root, and the ratio of root length to shoot length on Day 7. The seedling vigour index was calculated using the below formula according to Islam et al. [47].

Y = (a + b) c/100
(1)

where: Y is Seedling Vigour Index (SVI); a is the mean root length (cm); b is the mean shoot length (cm); and c is the percentage of germination rate (%).

### 2.6. Grain Quality Measurement

Duplicate barley samples were randomly taken from each treatment for quality measurement. A near infrared range (NIR) instrument (Infratec™ 1241 Grain Analyser, Foss, Melbourne, VIC, Australia) was utilised for analysing the grain quality. The functioning of this analyser is based on the measurement of transmission spectra of samples in the near-infrared region [48,49]. The instrument had an extended wavelength range of 570–1100 nm. A total of 10 subsamples of whole kernels were scanned for each grain sample. The parameters we assessed in this research included the total soluble protein content, moisture content (MC), colour, hardness, and thousand weight (TW) of grain. The colour results were reported as lightness, which was determined by L* values (0 = black and 100 = white). TW was estimated by weighing 1000 seeds in grams.

### 2.7. Statistical Analysis 

Data from the time-response bioassays were subjected to Probit analysis (IBM SPSS Statistics, version25.0). Time-mortality curves and LT_50_ and LT_95_ were generated. A one-way analysis of variance (ANOVA) and Tukey’s Honestly Significant Difference (HSD) test were employed to evaluate if there was a significant difference between the O_3_ treatments and control. The results were considered significant only when *p* ≤ 0.05. Origin 2019b was used for curve figures.

## 3. Results

### 3.1. Toxicity of O_3_ on R. dominica and T. castaneum Adults at Different Assessment Intervals

**Figure 1:**
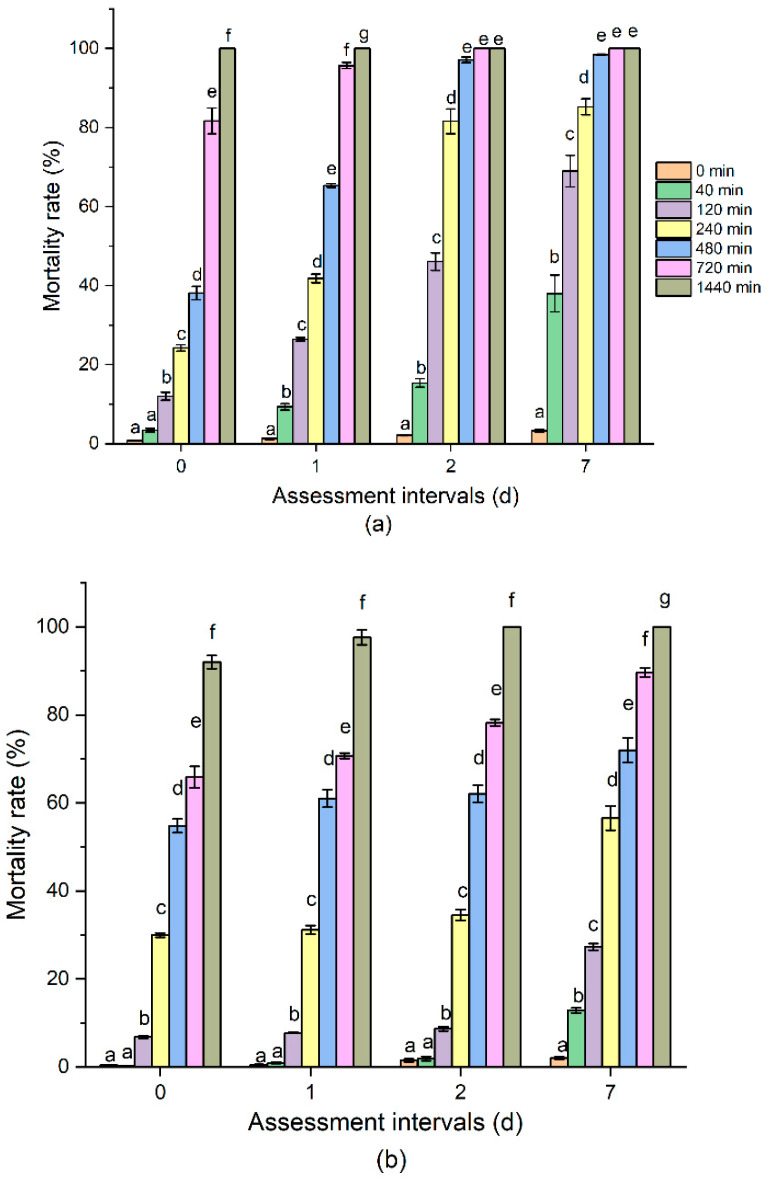
The mortality rate of (**a**) *Rhyzopertha dominica* and (**b**) *Tribolium castaneum* to different O_3_ exposure times at 0, 1, 2, and 7 days after O_3_ treatment. The bars with different letters (a–g) are significantly different (Tukey’s HSD test, *p* < 0.05).

The mortality rate of both the insect species increased with prolonged O_3_ exposure time and assessment interval (Figure 1). In terms of *R. dominica,* the mortality rate significantly increased with extended O_3_ exposure time. Adults of *R. dominica* and *T. castaneum* in barley could be knocked down immediately at 700 ppm for 2 h exposure, but 90% of them can be recovered after treatment. However, 1440 min of O_3_ treatment could kill 92% adults immediately, 97% at 1DAT, and 100% adults at 2 and 7DAT. *T. castaneum* displayed a similar tendency when subjected to different O_3_ exposure times and assessed at intervals after O_3_ treatment. The mortality rate was 81% at 0DAT, and then increased to 95% at 1DAT, and 100% at 2 and 7DAT when subjected to 960-min treatment. Time–mortality regression for the populations of *R. dominica* and *T. castaneum* that were exposed to O_3_ at different assessment intervals are represented in Table 1. A total of 700 ppm ozone of each treatment time (0, 40, 120, 240, 480, 960, and 1440 min) for the two species was conducted. The Pearson goodness-of-fit Chi-square (χ^2^) test showed that the probit model fit to the data was significant (*p* < 0.05), thus, a heterogeneity factor (χ^2^/df) was used in the calculation of the confidence limits. The LC_50_ values were estimated for each species at different assessment intervals. At 700 ppm O_3_, the median lethal time (LT_50_) of *R. dominica* on 0DAT was 472.91 (343–653.55) min and that of *T. castaneum* on 0DAT was 453.07 (228.14–769.72) min. The LT_90_ values for *R. dominica* and *T. castaneum* on 0DAT were 2419.93 (1465.95–6336.68) and 1823.12 (972.31–27,304.4) min, respectively. The data indicated that *R. dominica* was more tolerant to the O_3_ treatment. Additionally, the LT_50_ and LT_90_ of both insects at O_3_ treatment dramatically decreased on 1DAT, 2DAT, and 7DAT compared with 0DAT. The LC_50_ of *R. dominica* and *T. castaneum* on 7DAT were the lowest, which were 213.50 and 63.60 min, respectively. The relative potency for *R. dominica* and *T. castaneum* was 2.22 and 7.12, respectively. Our results indicated the delayed toxicity of O_3_ and that it is better to evaluate the O_3_ efficacy against these two species more than 1 day after treatment.

### 3.2. Toxicity of O_3_ Duration on All Life Stages of R. dominica and T. castaneum

The mortality rate of all life stages for *R. dominica* and *T. castaneum* that were exposed to different O_3_ durations is summarised in Table 2. Based on our preliminary study, 10 and 20 min O_3_ exposure could not kill the insects, so an O_3_ duration that was longer than 40 min was investigated. A total of 1440 min of O_3_ treatment could kill all stages of both species. The mortality rate of *R. dominica* adults significantly increased with extended O_3_ durations and reached 89.62% at 960-min exposure (F5, 12 = 29.334, *p* < 0.001), while *T. castaneum* reached 91.39% at 240-min exposure and 100% at 960-min treatment (F5, 12 = 209.197, *p* < 0.001). The mortality rate of both *R. dominica* and *T. castaneum* eggs, larvae and pupae also considerably increased with extended O_3_ durations and reached 83.33%, 91.67%, and 71.30% at 960-min exposure, while those of *T. castaneum* reached 81.11%, 100%, and 93.33% at 960-min treatment, respectively. In Table 2, the different letters in each life stage indicate significant mortality differences of the ozone treatment times between the two species. The results indicate that except for the egg stage, *T. castaneum* was more sensitive to the O_3_ treatment than *R. dominica*. Considerable variation was observed in susceptibility among all the life stages of *R. dominica* and *T. castaneum*. In terms of *R. dominica*, the adults and larvae were more susceptible to the O_3_ treatment than the pupae and eggs. The adults and larvae achieved 86.92% and 91.67% mortality with 960 min O_3_ treatment, however the pupae and eggs reached 74.30% and 83.33% mortality. Regarding *T. castaneum*, the observed mortality was 100% for the larvae and adults and 93.33% for the pupae, compared to 81.11% for the eggs with 960 min exposure.

### 3.3. Effect of O_3_ Treatment Duration on Germination of Barley

The germination rate is one of the most critical parameters that contributes to predicting final crop yield, which was measured 7 days after sowing. The effect of O_3_ on the germination of barley seeds are presented in Table 3. The seeds that were harvested in 2013/2014 and 2019/2020 are presented as old and new barley in this article. The germination rate of the old harvest seeds increased from 94.34% to 97.17% at 40 min ozonation, while that of the new barley seeds increased from 98.35% to 99.06% at 10 and 20 min ozonation. However, the long duration of the O_3_ treatment, such as 480, 960, and 1440 min has been observed to have a considerably negative effect on the germination rate of old harvest seeds. Regarding new seeds, the germination rate started to significantly decrease under 960 and 1440-min O_3_ exposure compared with the untreated seeds (*p* < 0.05). In both old and new barley seeds, O_3_ application times of 1440 min were accompanied by dramatical decreases in the germination rates to 14.39 % and 20.28%, respectively.

### 3.4. Effect of O_3_ Treatment Duration on Seedling Growth

**Figure 2:**
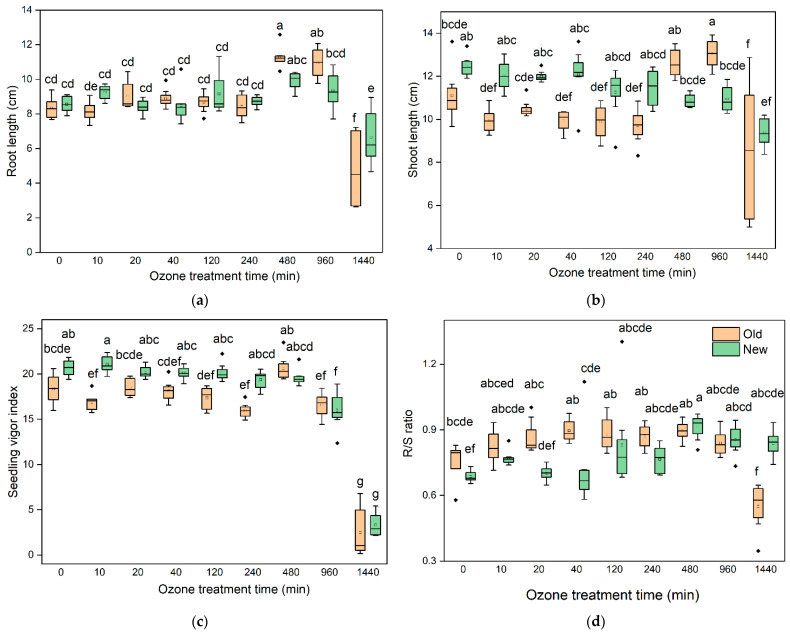
Changes in the morphological characteristics with O_3_ treatment duration on (**a**) barley root length; (**b**) barley shoot length; (**c**) seedling vigour index of barley seeds; (**d**) the ratio of root-to-shoot length. Significant differences (*p* < 0.05) between each treatment are indicated by letters. Groups do not have significant differences if they have identical marked letters. Statistical reliability of the differences was determined based on Tukey’s HSD test.

The effect of O_3_ on the morphological parameters (seedling length and vigour index) of barley seeds are presented in Figure 2. In terms of the total root length of new barley, there was no significant difference between 10, 20, 40, 120, 240, 480, and 960 min ozonation and the untreated samples. However, after 1440 min treatment, the shoot length decreased (6.65 cm) compared to the untreated samples (8.56 cm). For the old barley, 480 and 960 min O_3_ treatment significantly increased the root length to 11.28 and 10.95 cm from 8.34 cm, whereas the root length was significantly decreased to 4.79 cm at a longer exposure time (1440 min) compared with that of the control (8.34 cm) (Figure 2a). Regarding the shoot length, the new barley shoot length was not significantly inhibited by 10, 20, 40, 120, 240, 480, or 960-min O_3_ treatment, but was significantly inhibited at 1440 min ozonation. For the old barley seeds, there was a positive effect of O_3_ on the shoot length at 480- and 960-min treatment, which increased the shoot length to 12.61 cm and 13.05 cm compared with the untreated sample (11.11 cm). The shoot length was inhibited (8.49 cm) with the extension of O_3_ to 1440 min (Figure 2b).

The seedling vigour index (SVI) is an important parameter to reflect the potential field performance of different seeds. The SVI data is presented in Figure 2c. Regarding the new barley seeds, in comparison to the control, the SVI showed no significant difference under short duration ozonation, including 10, 20, 40, 120, 240, and 480 min. However, it dramatically decreased as the duration time increased to 960 and 1440 min (*p* < 0.05 value). In terms of the old seeds, only 1440 min O_3_ treatment significantly decreased the SVI compared with the untreated samples (*p* < 0.05). The ratio of root-to-shoot length (R/S ratio) in both the new and old barley seeds significantly decreased at 1440 min O_3_ treatment (*p* < 0.05). In addition, the R/S ratio in new barley seeds increased when they were subjected to 480 and 960 min exposure, however, in the old barley seeds there was no significant difference between 10, 20, 40, 120, 240, 480, or 960 min of O_3_ treatment and the control (Figure 2d).

### 3.5. Effect of O_3_ on Barley Grain Quality

**Figure 3:**
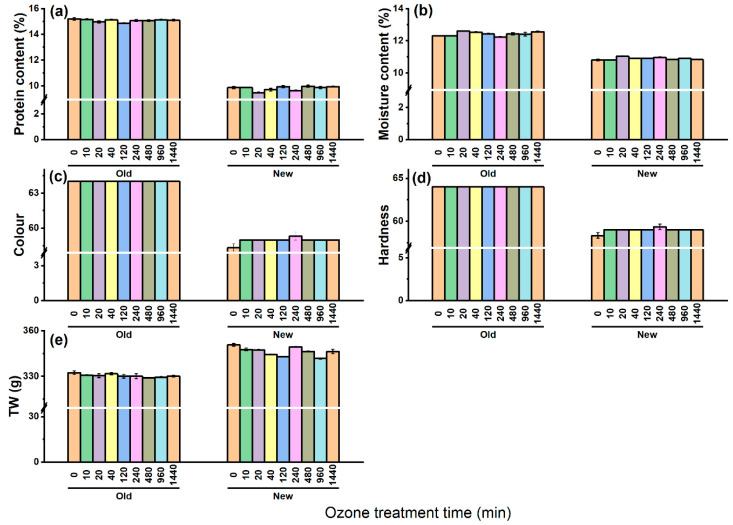
Effect of O_3_ duration on the quality of barley seeds. (**a**) The protein content; (**b**) moisture content; (**c**) colour; (**d**) hardness; (**e**) thousand seeds weight. There is no significant differences between each ozonetreatments based on Tukey’s HSD test.

The soluble protein content of old and new barley seeds was 15.2% and 9.9%, respectively (Figure 3a). It can be observed that there was no significant effect of O_3_ on the soluble protein content in both samples, irrespective of the age. The moisture content of the old and new barley varied from 12.2% to 12.6% and 10.7% to 11%, respectively, and showed no change with increasing O_3_ duration in both the sample groups (Figure 3b). From Figure 3c, the old barley had a higher lightness value than the new barley. The colour of both the new and old barley was not affected by the O_3_ duration. As can be seen from Figure 3d, the hardness values for the old and new barley were 55.3 and 45.3, respectively. There was a less apparent trend towards increasing hardness of both the old and new seeds, characterised by small fluctuations up to 58 and 47.6. Generally, the TW of both the old and new barley was not considerably impacted by extended periods of O_3_ treatment. In terms of the old barley, the TW was decreased from 33.2 to 32.9 g, while the new barley was reduced from 35.1 to 34.2 g (Figure 3e). Overall, the value of the soluble protein content, moisture content, colour, and hardness of old barley were higher, and only thousand weight (TW) was lower than that of the new barley seeds (Figure 3).

## 4. Discussion

The results suggest that O_3_ has efficacy against both *R. dominica* and *T. castaneum*, with the toxicity data indicates that mortality occurs in a relatively short interval and increased with prolonged O_3_ treatment. Sufficient exposure time is required to kill insects since disinfestation action of O_3_ takes place mainly at the surface of the grain, unless saturation of the grain is reached [44]. It was observed that 960-min O_3_ treatment could kill 65% and 81% of *R. dominica* and *T. castaneum*, even if assessed immediately after O_3_ treatment. Lower exposure time led to a higher mortality rate of insects using longer assessment intervals. Thus, both exposure times and mortality assessment intervals should be considered to evaluate pesticide efficacy.

The assessment intervals of detecting insect mortality to pesticides should be varied based on insecticide mode of actions, namely 1–2 days for fast-acting insecticides, and 7–14 days for slow-acting insecticides. Our results indicated the delayed toxicity of O_3_ since the mortality of both species increased up to seven days, which is in accordance with Subramanyam et al. and Holmstrup et al. [50,51]. The mechanism of ozone killing insects is still elusive. There are two main hypothesised mechanisms. The first one is related to the respiratory rate. The respiratory system of insects is considered to be the first site of action for O_3_, as it enters insects through spiracles causing disrupted respiration in insects. Discontinuous gas exchange could lower insect metabolisms causing insect death [52]. The second hypothesis is based on strong the oxidative properties of O_3_, which could cause oxidative damage of essential components resulting in DNA strand breaks and cell membrane oxidation [53]. Another possible reason is that gene expression and damage to DNA exposures do not occur immediately after O_3_ exposure, but in the post-exposure period [51].

Toxicity data indicated a noticeable difference in the susceptibility between *R. dominica* and *T. castaneum*, especially at different life stages. In general, all life stages except for eggs of *R. dominica* have a higher tolerance to O_3_ than *T. castaneum*. In addition, longer O_3_ exposure times at 700 ppm were required for eggs and pupae of both species to reach higher mortality compared with larvae, which agrees with previous research [36,54]. Distinct responses of life stages to O_3_ could be attributed to their respiration rates. The respiratory system of insects is considered as the first site of action for O_3_. On the basis of phosphine studies [55], we suggest that the low respiration rate of pupae and eggs might reduce the O_3_ uptake resulting in greater tolerance [56]. Furthermore, the majority of the outer layer of eggs and pupae are made of lipids, sometimes covered with a waxy coat which could provide an additional barrier against O_3_.

The current results indicate that, to obtain 100% mortality of all stages of both species, the required exposure time of O_3_ at 700 ppm was 1440 min. The short duration O_3_ treatment did not significantly influence the germination ability in either the old or new barley. However, with increasing duration, the germination ability was strongly impacted. These observations are in agreement with Wu et al. [57], who suggested that the germination ability of stored wheat was damaged with prolonged O_3_ treatment. Peroxide or superoxide accumulation, which is elicited by O_3_, can damage antioxidant mechanisms, reduce the activity of related enzymes, and disturb macromolecule biosynthesis, resulting in suppression of the germination process. This is problematic, as quality of barley is crucial in determining its end utilisation. For example, the germination rate of barley seed must exceed 95% for malting [58]. Therefore, a high seed germination ability plays a pivotal role in improving crop production and shortening the manufacturing costs of the malting process. Some studies have indicated that moderate duration ozonation can accelerate the germination of tomato seeds because reactive oxygen species (ROS) that are produced by O_3_ promote cell signalling [59]. The ROS break seed dormancy and promote germination by regulating hormone levels and reducing abscisic acid levels [60]. In this study, the germination ability of two samples of different ages was enhanced under moderate ozonation time, especially for old seeds, which increased from 94.34% to 97.17% at 40 min O_3_ treatment, improving the germination ability of aging barley to meet the malting barley grade. Further research needs to be conducted, specifically utilising seeds with a lower germination ability to explore if moderate O_3_ processing time can significantly increase the germination ability.

This study also suggested that prolonged O_3_ exposure (1440 min) was unfavourable for seedling growth. Both the root and shoot length were decreased with extending O_3_ duration to 1440 min, which is consistent with reports that maize seedling length decreased with increasing O_3_ processing time [61]. Additionally, the root length of both old and new barley significantly increased under moderate O_3_ processing time. Seedling growth is a cell division and expansion process, and endogenous reactive oxygen species (ROS), such as hydrogen peroxide (H_2_O_2_) that are induced by O_3_, play essential roles in cell growth [62]. The root-to-shoot ratio is a good indicator of the overall health and physiology of plants. It is generally genetically fixed for each cultivar but can be modified by environmental factors [63]. The ratio of the root length to the shoot length increased when the seeds were subjected to longer O_3_ exposure time, signifying that plants reduce shoot growth and enhance root growth in response to O_3_ stress. Alexander et al. [64] suggested that the root-to-shoot ratio of spring wheat was increased from increasing O_3_ concentration. Plants apportioning more biomass to roots and less to stems when they encounter unfavourable conditions has been previously established [65].

Quality characteristics including the soluble protein content, colour, and TW may affect the final market price of grain. In this research, different O_3_ treatment times did not influence the soluble protein content, moisture content, TW, and colour of boththe old and new seeds, which closely correlated with previous studies [66,67]. Seed storage is one of the most important factors that affect seed quality. Seed reserves are lost during storage, as manifested by the lower TW of old seeds. The total soluble protein was higher in the old seeds compared with the new ones, consistent with Lozano et al. [68] who reported that the total soluble protein and amino acid content of *Jatropha curcas* L. seeds increased by 160% and 67% during storage, respectively. A possible explanation could be the consequence of soluble protein or amino acid that is converted from storage proteins to maintain respiration and respond to stress during storage [69]. As the energy resources are used by the embryo during germination, organic compounds in the seeds continually reduce due to respiration processes after harvest, resulting in an energy deficiency during germination and early seedling development. This explains why the old seeds in this study appeared to have a lower germination ability and seedling growth capacity compared to the new seeds. Additionally, old seeds tend to be more susceptible to O_3_ stress. The germination rate of the old seeds significantly reduced at 480 min ozonation, whereas that of the new barley did not significantly decrease until 960 min exposure.

Although the morphological characteristics and germination were adversely influenced by excess ozone exposure, that can be regulated to achieve desired outcomes if proper measures are taken. For instance, if the barley seeds are utilised for livestock feeding and human consumption, it is necessary to supply sufficient ozone treatment to eliminate pests rather than achieve high germination ability. Most farmers grow barley for sale as malting barley where germination ability and other malting quality parameters could also be maintained and even improved based on the proper application of ozone. This research found that short ozone exposure could enhance the germination ability of aging seeds to meet malting barley requirements. All these studies make it evident that ozone has great potential to improve the functionalities of grain products while ensuring food safety.

## 5. Conclusions

More than two days after ozone treatment should be considered as the endpoint to evaluate insects’ mortality rates due to delayed toxicity of O_3_. C × t product 36 mg h/L (700 ppm × 24 h) of ozone offered complete mortality for all stages of the two species of tested insects at an endpoint of dead, which is highly toxic than phosphine and enthyl formate and without quality parameters including soluble protein content, moisture content, colour, hardness, and TW. The pupae and eggs were more tolerant to O_3_ than the larvae and adults. Moderate ozone treatment time such as 20 min and 40 min increased the germination ability to greater than 95%, however, the germination ability and seedling growth of both the old and new barley seeds were impaired due to long exposure to O_3_ (1440 min), especially for the longer storage seeds. Overall, the data demonstrated that the multiple effects of ozone treatment on the parameters of barley seeds could be utilised to control the quality attributes of final food products, thus, it is imperative to select an optimal O_3_ exposure time to achieve the desired function, such as malting, animal feeding, and human consumption. Future investigations are needed to evaluate the O_3_ efficacy against insects in large-sized silos, as well as elaborate on how metabolites in barley seeds shift in response to O_3_ treatment.

## Figures and Tables

**Table 1:** Regression analysis for mortality rate of *Rhyzopertha dominica* and *Tribolium castaneum* at different assessment intervals. 700 ppm ozone of each treatment time (0, 40, 120, 240, 480, 960, and 1440 min) for two species were conducted.

Insects	DAT ^†^	Slope ^‡^ ± SE	Intercept ± SE	Relative Median Potency *	LT_50_ (95% CI ^§^) (min)	LT_95_ (95% CI) (min)	χ^2^^¶^	df
*R. dominica*	0	2.32 ± 0.05	−6.21 ± 0.15	1.00	472.91 (343.04–653.55)	2419.93 (1465.95–6336.68)	118.02	6
	1	2.48 ± 0.06	−6.48 ± 0.15	1.15	410.12 (278.81–587.64)	1887.41 (1130.49–5594.71)	162.50	6
	2	2.74 ± 0.06	−7.01 ± 0.17	1.25	378.37 (265.87–515.29)	1510.96 (976.73–3617.63)	138.20	6
	7	2.06 ± 0.05	−4.80 ± 0.12	2.22	213.50 (146.17–291.40)	1342.06 (843.67–3033.99)	95.58	6
*T. castaneum*	0	2.72 ± 0.07	−7.23 ± 0.20	1.00	453.07 (228.14–769.72)	1823.12 (972.31–27304.4)	322.13	6
	1	2.31 ± 0.06	−5.54 ± 0.14	1.80	252.04 (143.69–378.75)	1300.80 (747.48–4884.78)	198.01	6
	2	2.85 ± 0.07	−5.88 ± 0.16	3.91	115.79 (92.92–139.38)	437.12 (341.45–620.62)	40.70	6
	7	2.30 ± 0.07	−4.15± 0.14	7.12	63.60 (49.06–78.09)	329.40 (256.77–463.99)	27.22	6

^†^ Day after O^3^ treatment; ^‡^ Mean ± standard error; * Relative potency = LC _50_ of 0 DAT/LC_50_ of another DAT (1, 2, 7); ^§^ Confidence interval; ^¶^ Chi-square value for goodness-of-fit of probit model to data.

**Table 2:** Efficacy of O_3_ duration at 700 ppm on all life stages of *Rhyzopertha dominica* and *Tribolium castaneum*.

Life Stage	O_3_ Treatment Time (min)	*Rhyzopertha dominica*	*Tribolium castaneum*
		Mortality Rate (%) ^†^	Mortality Rate (%)
Egg	0	0.00 ± 0.00 ^g^	1.67 ± 1.67 ^g^
	40	18.33 ± 3.33 ^fg^	17.92 ± 1.5 ^fg^
	120	38.33 ± 3.33 ^ef^	33.33 ± 3.85 ^ef^
	240	68.33 ± 4.41 ^bcd^	49.01 ± 5.21 ^de^
	480	70.63 ± 5.23 ^bc^	61.52 ± 9.34 ^cd^
	960	83.33 ± 6.01 ^ab^	81.11 ± 2.22 ^abc^
	1440	100.00 ± 0.00 ^a^	100.00 ± 0.00 ^a^
Larvae	0	3.33 ± 1.92 ^g^	3.70 ± 0.93 ^g^
	40	26.67 ± 3.33 ^f^	25.56 ± 2.94 ^f^
	120	53.33 ± 6.67 ^e^	51.38 ± 2.01 ^e^
	240	60.42 ± 5.51 ^de^	82.88 ± 1.93 ^bc^
	480	70.83 ± 2.08 ^cd^	99.67 ± 0.33 ^a^
	960	91.67 ± 2.08 ^ab^	100.00 ± 0.00 ^a^
	1440	100.00 ± 0.00 ^a^	100.00 ± 0.00 ^a^
Pupae	0	0.00± 0.00 ^g^	2.78 ± 1.60 ^g^
	40	12.95 ± 2.50 ^fg^	24.39 ± 3.43 ^ef^
	120	28.62 ± 3.10 ^de^	51.82 ± 4.30 ^cd^
	240	44.21 ± 4.89 ^c^	64.55 ± 2.92 ^b^
	480	63.82 ± 4.20 ^b^	74.24 ± 2.98 ^b^
	960	74.30 ± 1.86 ^b^	93.33 ± 3.33 ^a^
	1440	100.00 ± 0.00 ^a^	100.00 ± 0.00 ^a^
Adults	0	2.06 ± 0.32 ^h^	3.21 ± 0.61 ^gh^
	40	12.85 ± 0.58 ^g^	38.00 ± 8.09 ^e^
	120	27.29 ± 0.77 ^f^	68.97 ± 6.90 ^c^
	240	56.57 ± 2.74 ^d^	85.24 ± 3.51 ^b^
	480	71.95 ± 2.81 ^c^	98.45 ± 0.29 ^a^
	960	89.62 ± 1.06 ^ab^	100.00 ± 0.00 ^a^
	1440	100 ± 0.00 ^a^	100 ± 0.00 ^a^

^†^ Mean ± standard error. Different letters in each life stage indicate significant mortality differences at ozone treatment times between the two species (*p* < 0.05, Tukey’s HSD test).

**Table 3:** Effect of O_3_ duration at 700 ppm on the germination rate of old harvest and newly harvest barley seeds.

O_3_ Treatment Duration (min)	Germination Rate (%)	
	Old (2013/2014)^ †^	New (2019/2020)
0	94.34 ± 1.67 ^ab^	98.35 ± 0.56 ^a^
10	94.11 ± 0.97 ^ab^	99.06 ± 0.36 ^a^
20	95.28 ± 0.87 ^ab^	99.06 ± 0.36 ^a^
40	97.17 ± 1.01 ^a^	98.59 ± 0.59 ^a^
120	95.28 ± 1.01 ^ab^	98.82 ± 0.79 ^a^
240	84.21 ± 1.12 ^bc^	96.23 ± 0.71 ^a^
480	86.32 ± 0.93 ^c^	94.34 ± 0.94 ^a^
960	68.16 ± 1.61 ^d^	78.54 ± 2.71 ^b^
1440	14.39 ± 4.55 ^e^	20.28 ± 1.84 ^c^

^†^ Mean ± standard error. Means in the same column with different letters (a–e) are significantly different at *p* < 0.05 following Tukey’s HSD test.

## Data Availability

Data is contained within the article.
